# Supplementary material for: N-dihydrogalactochitosan reduces mortality in a lethal mouse model of SARS-CoV-2
Source: PLoS One. 2023 Aug 8;18(8):e0289139. doi: 10.1371/journal.pone.0289139 (PMC10409267; doi:10.1371/journal.pone.0289139)
Supplement: S1 File — (PDF) [file pone.0289139.s001.pdf]

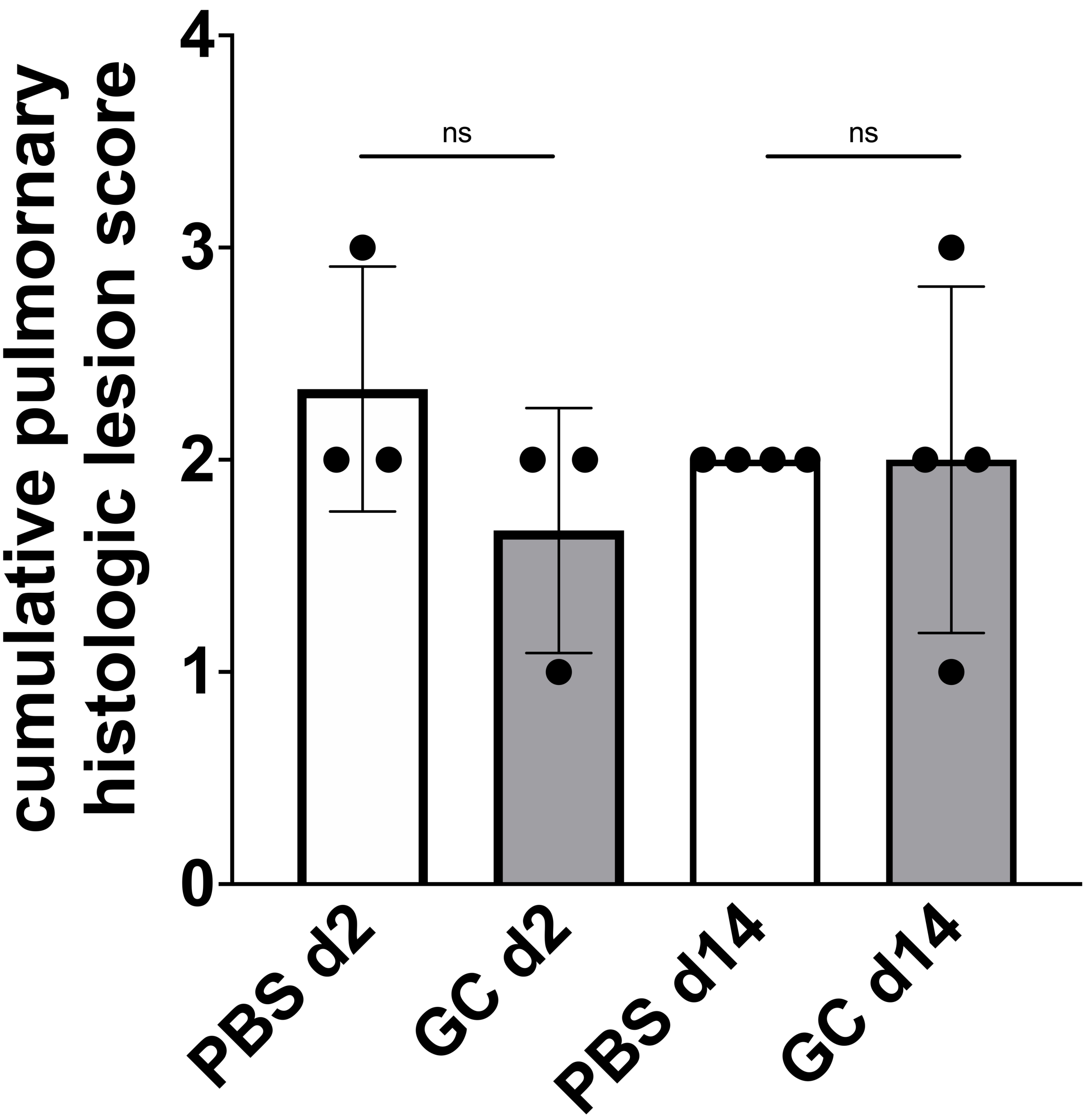

Supplementary Fig. 1. **GC administered intranasally does not produce histopathologic changes in murine lung tissues**. Six-week old male and female mice were treated intranasally at 3 intervals with GC or PBS following the same schedule used for mice in the SARS-CoV-2 cohorts. Mice were euthanized 2 or 14 days after the last treatment. Cumulative lung lesion scores were determined using the scoring scale

in Supplementary Table 2. A comprehensive list of lesions detected in each animal is shown in Supplementary Table 2. ns is not significant, Mann Whitney test (P=0.6).

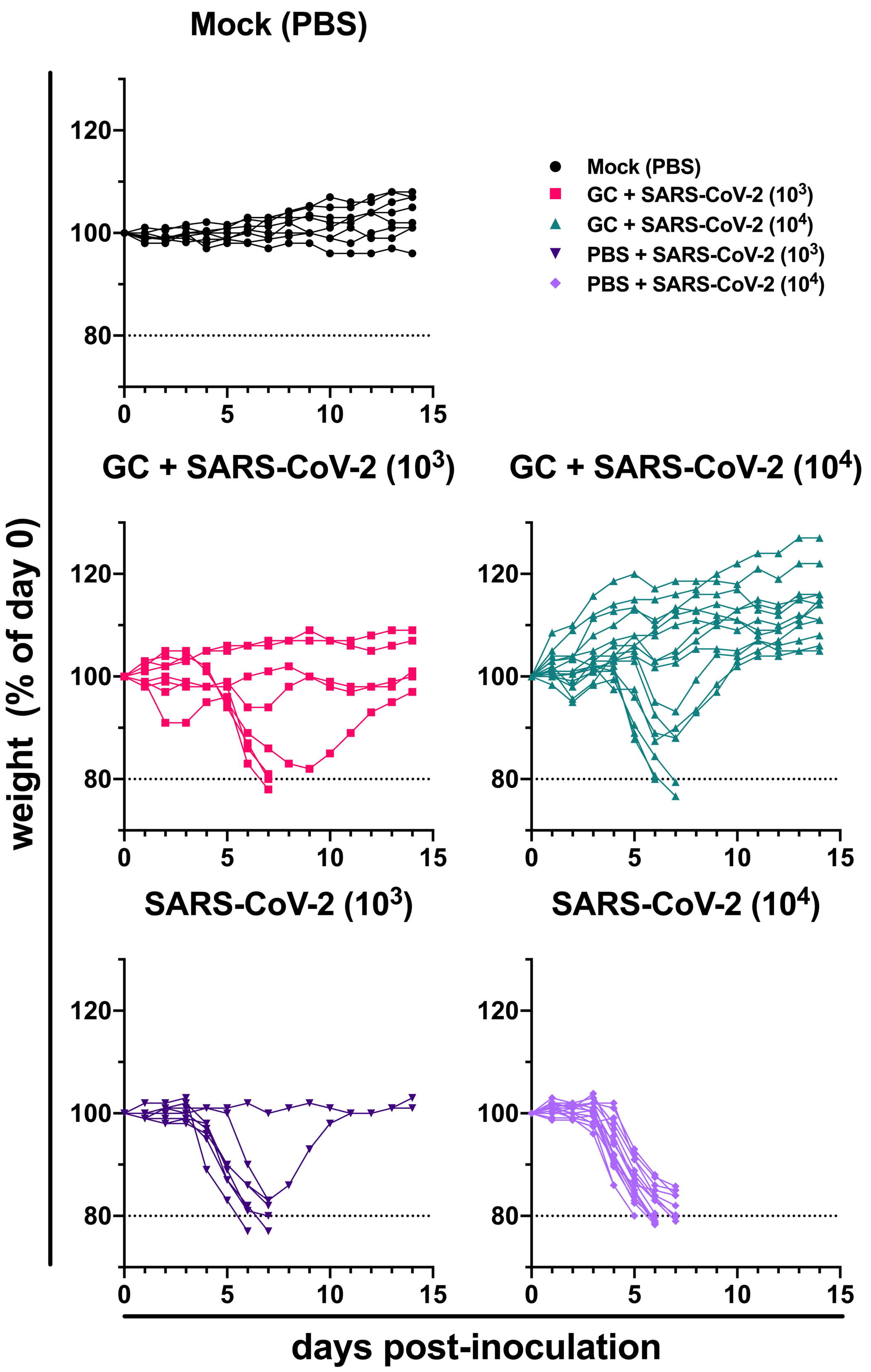
Mock (PBS)
GC + SARS-CoV-2 (10^3)
GC + SARS-CoV-2 (10^4)
SARS-CoV-2 (10^3)
SARS-CoV-2 (10^4)
Mock (PBS)
GC + SARS-CoV-2 (10^3)
GC + SARS-CoV-2 (10^4)
PBS + SARS-CoV-2 (10^3)
PBS + SARS-CoV-2 (10^4)
weight (% of day 0)
days post-inoculation
120
100
80
0
5
10
15

11

Supplementary Fig. 2: N-dihydrogalactochitosan protects mice from SARS-CoV-2 weight loss. Each line shows individual mouse weight as a percentage of their starting weight at the time of inoculation.

Supplementary Table 1: Lung Histologic Scoring in PBS and GC treated mice. Rows show quantitative histologic scoring in H&E treated lung tissue from 6-week old male and female K18 mice treated with PBS or GC treated mouse euthanized 2 or 14 days after administration of 30 ul 0.75% GC intranasally on day 0. The cumulative lesion score was determined using the scale in Supplementary Table 2. Sections were visualized by a board certified pathologist who was blinded to treatment group.

			**Pulmonary histopathologic findings**						**Cumulative lesion score**	**Notes**
**Treatment Animal**	**Sex**	**Day euthanized post treatment**	**Edema**	**Hyaline membranes**	**Inflamma tion**	**Thrombi**	**Hemorrhage**	**Type II hyperplasia**		
PBS 1	F	2	1	0	1	0	0	1	3	
PBS 2	M	2	0	0	1	0	0	1	2	
PBS 3	F	2	1	0	1	0	0	0	2	Focal foreign body granuloma
PBS 4	M	14	1	0	1	0	0	0	2	
PBS 5	M	14	1	0	0	0	0	1	2	
PBS 6	F	14	1	0	0	0	0	1	2	
PBS 7	F	14	0	0	1	0	0	1	2	
GC 1	M	2	1	0	0	0	0	0	1	
GC 2	M	2	1	0	0	0	0	1	2	
GC 3	F	2	1	0	0	0	0	1	2	
GC 4	M	14	1	0	1	0	0	1	3	
GC 5	F	14	1	0	0	0	0	1	2	
GC 6	F	14	0	0	1	0	0	0	1	
GC 7	M	14	1	0	0	0	0	1	2	

Supplementary Table 2: Lung histopathology scoring criteria.

Score		Description
0	none	Within normal limits or rare, scattered lymphocytic infiltrates not observed in control animals but significance questionable (could be background lesion or variation of normal).
1	minimal	Minimal mononuclear inflammation affecting less than 2% of the section. Inflammatory leukocyte infiltration is limited to a perivascular and/or peribronchiolar distribution with no evidence of alveolar or vascular injury.
2	mild	Mild peribronchiolar/perivascular inflammation which may also expand alveoli/alveolar septa, composed primarily of macrophages and lymphocytes (+/- scattered neutrophils); increased alveolar macrophages; alveolar septal architecture largely intact; affects 2-10% of the section; and/or scattered alveolar hemorrhage/fibrin/edema; and/or scattered atypical/multinucleated syncytial cells.
3	moderate	Moderate bronchointerstitial and perivascular inflammation, increased alveolar macrophages, and/or alveolar hemorrhage/fibrin/edema (characterized as above); and/or alveolar damage (characterized by type I pneumocyte necrosis or loss with replacement by hyaline membranes, fibrin, edema, and/or necrotic debris); and/or reparative/regenerative changes (type II pneumocyte hyperplasia, atypical/multinucleated syncytial cells, or fibrosis); lesions affect 10-25% of the section.
4	severe	As above but more widespread inflammation, hemorrhage/fibrin/ edema, and/or alveolar damage/loss of normal septal architecture; and or regenerative changes affecting greater than 25% of the section.
+1		Add 1 point if: Greater than 25% of inflammatory cells are neutrophils; there is significant necrotizing vasculitis, endotheliitis or microthrombi; or if there is significant bronchiolitis, airway epithelial necrosis or hyperplasia.

Supplementary Table 3: Nasal concha histopathology scoring criteria.

Score	Degree	Nasal Cavity
**0**	none	Within normal limits or rare, scattered lymphocytic infiltrates and/or rare foci of mucus, proteinaceous fluid, sloughed epithelium and/or free RBCs not observed in control animals but significance questionable (could be background lesion or variation of normal)
**1**	minimal	As above with minimal mononuclear submucosal and/or perivascular inflammation
**2**	mild	Mild rhinitis characterized by mucosal/submucosal primarily mononuclear inflammation (+/- low numbers of neutrophils) which may extend into the nasal cavity (along with sloughed epithelial cells and small amounts of mucus/fibrin); overall normal/intact nasal architecture.
**3**	moderate	Moderate rhinitis characterized by a mixed inflammatory mucosal/submucosal infiltrate and/or nasal cavity exudate composed of neutrophils, macrophages, and lymphocytes +/- mild to moderate hemorrhage, fibrin, edema and/or necrotic debris.
**4**	severe	As above but with more widespread and severe inflammation and/or mucosal erosion/necrosis.
